# Supplementary material for: Efficacy of FFP3 respirators for prevention of SARS-CoV-2 infection in healthcare workers
Source: eLife. 2021 Nov 16;10:e71131. doi: 10.7554/eLife.71131 (PMC8635983; doi:10.7554/eLife.71131)
Supplement: Supplementary file 1. [file elife-71131-supp1.docx]

**Supplementary File 1: Additional data tables**

**Case numbers and HCW days for critical care wards**

| **Week** | **Week start** | **Critical care cases** | **Critical care HCW days** |
| --- | --- | --- | --- |
| 1 | 02/11/2020 | 1 | 994 |
| 2 | 09/11/2020 | 0 | 994 |
| 3 | 16/11/2020 | 1 | 994 |
| 4 | 23/11/2020 | 1 | 994 |
| 5 | 30/11/2020 | 0 | 994 |
| 6 | 07/12/2020 | 3 | 994 |
| 7 | 14/12/2020 | 1 | 994 |
| 8 | 21/12/2020 | 3 | 994 |
| 9 | 28/12/2020 | 3 | 994 |
| 10 | 04/01/2021 | 2 | 1001 |
| 11 | 11/01/2021 | 0 | 1043 |

**Roles of HCWs with SARS-CoV-2 infection according to ward type**

| **Job titles** | **Red (%)** | **Green (%)** | **Total (%)** |
| --- | --- | --- | --- |
| Nurse | 17 (63.0) | 74 (52.9) | 91 (54.5) |
| HCA | 6 (22.2) | 35 (25.0) | 41 (24.6) |
| AHP | 0 (0) | 17 (12.1) | 17 (10.2) |
| Domestic | 2 (7.4) | 8 (5.7) | 10 (6.0) |
| Doctor | 1 (3.7) | 5 (3.6) | 6 (3.6) |
| Admin | 1 (3.7) | 1 (0.7) | 2 (1.2) |
| Total | 27 | 140 | 167 |
